# Cancer-Risk Module Identification and Module-Based Disease Risk Evaluation: A Case Study on Lung Cancer

**DOI:** 10.1371/journal.pone.0092395

**Published:** 2014-03-18

**Authors:** Xu Jia, Zhengqiang Miao, Wan Li, Liangcai Zhang, Chenchen Feng, Yuehan He, Xiaoman Bi, Liqiang Wang, Youwen Du, Min Hou, Dapeng Hao, Yun Xiao, Lina Chen, Kongning Li

**Affiliations:** College of Bioinformatics Science and Technology, Harbin Medical University,Harbin,Hei Longjiang Province, China; University of Georgia, United States of America

## Abstract

Gene expression profiles have drawn broad attention in deciphering the pathogenesis of human cancers. Cancer-related gene modules could be identified in co-expression networks and be applied to facilitate cancer research and clinical diagnosis. In this paper, a new method was proposed to identify lung cancer-risk modules and evaluate the module-based disease risks of samples. The results showed that thirty one cancer-risk modules were closely related to the lung cancer genes at the functional level and interactional level, indicating that these modules and genes might synergistically lead to the occurrence of lung cancer. Our method was proved to have good robustness by evaluating the disease risk of samples in eight cancer expression profiles (four for lung cancer and four for other cancers), and had better performance than the WGCNA method. This method could provide assistance to the diagnosis and treatment of cancers and a new clue for explaining cancer mechanisms.

## Introduction

Cancer is caused by aberration of multiple genes, and thus its pathogenesis is very complex and inconclusive [1], [2], [3]. Cancer-related genes possess diverse functions [4], [5], while genes with similar functions are likely to be co-expressed [6], [7] and located in neighboring areas (known as network modules) [8], [9] in biological networks. The modules reveal the mechanism of multiple genes underlying the disease and evaluate the risk of the disease. Effective identification of cancer risk modules can assist cancer researches [10], [11], [12], [13].

Disease risk of cancer-related modules calculated from a specific biological background can be a significant measure for clinical prediction of cancer diagnosis [14], [15], [16], [17], [18]. Several computational approaches have been developed for the disease risk module analysis, including detection of differentially correlated gene clusters and gene-specific analysis based the co-expression network [19], [20], [21], [22]. For example, weighted gene co-expression network analysis (WGCNA) is a mature technique and identifies gene modules as candidate biomarkers or therapeutic targets based on the co-expression network [23], [24]. WGCNA has been used to study complex diseases, such as metabolic syndrome [25], schizophrenia [26], and heart failure [27]. The expression activities of disease risk modules were (induced or repressed) different among clinical conditions (in tumor progress)[14].

Furthermore, it is feasible to identify cancer risk modules from co-expression networks using network-based methods. The analysis of gene co-expression networks shows that genes within the same modules appear to have similar expression patterns, share common regulatory mechanisms [28], [29], [30], and thus have strong associations with specific biological functions that determine the behaviors or phenotypes of cells [31], [32]. Modules derived from co-expression network were organized into a higher-order structure correlated with clinical characteristics, which provided insights into the underlying biology of glioma [33]. Four modules of ovarian cancer from a co-expression network were distinguished to be significantly associated with biological processes such as cell cycle and DNA replication in Gene Ontology (GO) categories[34]. The co-expression modules associated with T-helper differentiation and TGF-beta pathways improved clinical outcome of hormone-insensitive breast cancers after treatment [35]. Moreover, sample signatures/labels considered in evaluation of cancer-related risk modules would offer a new clue for revealing the mechanisms of diseases [36]. Researches have revealed that it is necessary to explore the relationships between gene functions and disease risks [37], [38]. The co-expression networks taking into account of biological functions would be more robust and authentic [39], [40], and the modules obtained from these networks could better reflect the function information of the diseases.

**Figure 1:**
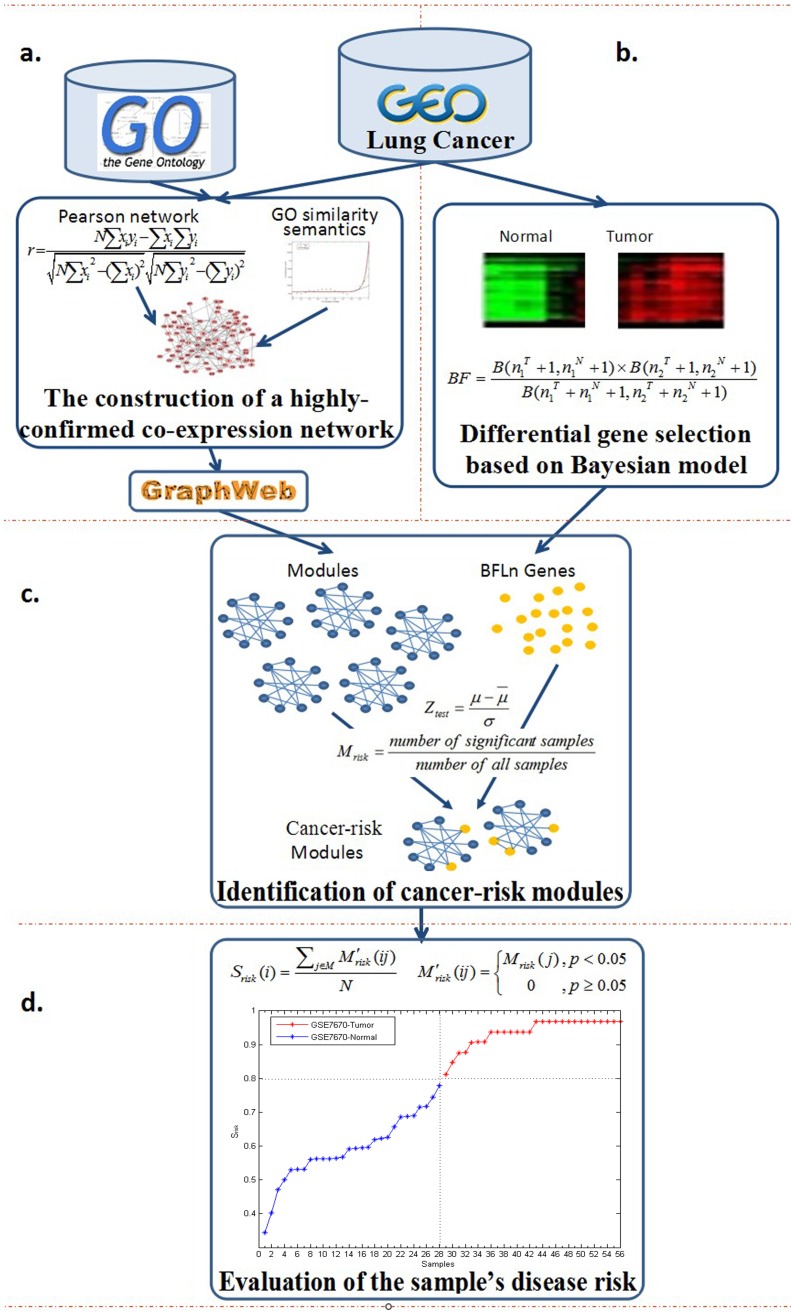
Cancer-risk Modules Identification and Module-based Disease risk Evaluation.

In this paper, a new method was proposed to identify cancer-risk modules and evaluate the module-based disease risks of samples. A highly-confident co-expression network with functional similarity information was first constructed by using expression profiles in lung cancer, and then candidate modules were identified. The cancer risks of the modules were scored by introducing sample labels, then the significant cancer-risk modules were screened out by randomized trials. Finally, the disease risks of samples were evaluated based on the cancer-risk modules. These modules were expected to provide evidence for disease diagnosis, treatment and clinical analysis in the future. Identification of cancer-risk modules and evaluation of module-based disease risks were performed in the following steps (Figure 1).

## Materials and Methods

### Materials

Cancer gene expression data were obtained from the Gene Expression Omnibus(GEO, http://www.ncbi.nlm.nih.gov/geo/)[37]. Here, our research was based on the profile GSE7670 [41]in GPL96 including 20,995 genes of 56 samples (28 lung cancer patients and 28 normal controls), for which patients underwent surgery for lung cancer at the Taipei Veterans General Hospital. These expression profiles (GSE10072, GSE21933, GSE27262, GSE40791, GSE14520, GSE15781, GSE20437, GSE26126) (Table 1) with disease and normal samples were used to analyze the robustness of our method and compare with the WGCNA method. Gene function information was obtained from Gene Ontology (GO, http://www.geneontology.org/) [42], updated to May 2011. Protein interaction information (95537 high-confidence interactions between 12359 genes) was downloaded from iRefWeb (http://www.wodaklab.org/iRefWeb/) [43], updated to April 13, 2012 of the 9th version. The information of 1824 protein complexes was obtained from Munich Information Center for Protein Sequences (MIPS, http://mips.helmholtz-muenchen.de/genre/proj/corum, Corum Release February 2012 available).

**Table 1:** The number of the tumor samples and the normal samples in the expression profiles.

	GSE10072	GSE21933	GSE27262	GSE40791	GSE14520	GSE15781	GSE20437	GSE26126
**Cancer**	Lung Cancer				Liver Cancer	Colon Cancer	Breast Cancer	Prostate Cancer
**GPL**	GPL96	GPL6254	GPL570	GPL570	GPL3921	GPL2986	GPL96	GPL8490
**Tumor**	58	21	25	94	64	13	18	181
**Normal**	49	21	25	100	64	10	15	12

#### a. The construction of a highly confident co-expression network

A method was introduced to create a highly confident co-expression network by taking both co-expression correlation and functional similarity. This method was performed as follows:

First, the Pearson correlation coefficient [44] *r* was used to represent the co-expression relationship between every pair of genes and calculated as follows:
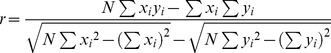
where *N* is the number of samples in an expression profile, *x_i_* and *y_i_* are the expression levels of genes *x* and *y* in the *i*-th sample.

Second, GO semantic similarity was used to represent the functional similarity between every pair of genes [45].

(1) The similarity score of GO term A was defined as:
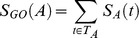





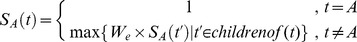





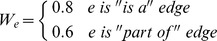
where 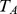 includes term A and all its parent terms; 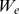 is the weight of edge; and it is 0.8 for ‘is-a’ relationship and 0.6 for ‘part-of’ relationship.

(2) The semantic similarity between term A and term B, 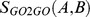, was calculated as follows:
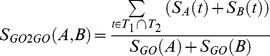



A gene's functions were considered as a set of GO terms in Gene Ontology. Thus, functions of genes G1 and G2 corresponded to GO sets 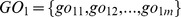
and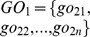, *m* and *n* are the number of terms in GO1 and GO2 respectively.

(3) The semantic similarity between G1 and G2 was defined as:
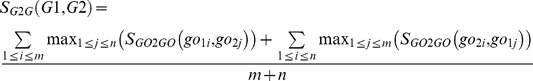



The robust gene pairs were retained by the function similarity. Therefore, a highly-confident co-expression network was constructed by analyzing the Pearson correlation coefficient and GO semantic similarity.

#### b. Differential gene selection based on Bayesian model

A Bayesian model [46], [47] was used to screen the differential genes. Bayesian approaches compare the probability of an association between a gene expression and a disease to the probability given no such association. The formula was as follows:
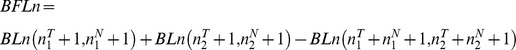
where *n_1_^T^*, *n_2_^T^*, *n_2_^N^* and *n_2_^N^* are the number of samples (tumor/normal and high/low expression) for one gene (Table 2). B denotes the Beta function, defined by

**Table 2:** The number of samples (tumor/normal and high/low expression) for one gene.

	+	−	Total
T	*n_1_^T^*	*n_2_^T^*	*n_1_^T^ + n_2_^T^*
N	*n_1_^N^*	*n_2_^N^*	*n_1_^N^ + n_2_^N^*
Total	*n_1_^T^ +n_1_^N^*	*n_2_^T^ + n_2_^N^*	*n_1_^T^ + n_2_^T^ + n_2_^N^ + n_2_^N^*

T represents tumor samples and N for normal ones, and “+” stands for high expression (above-average) and “-”for low expression (below-average). *n_1_^T^* and *n_2_^T^* refers to the number of tumor samples with high expression and low expression, and *n_1_^N^* and *n_2_^N^* for the number of normal ones with high expression and low expression.



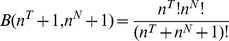



*BLn* is the log value of B.

When *BFLn*>0, there was relationship between a disease and gene expression; when *BFLn*<0, no relationship.

A randomized test was designed to calculate the significance of *BFLn* by stochastically disturbing *n_1_^T^*, *n_2_^T^*, *n_2_^N^* and *n_2_^N^* and retaining stable sum; after 10,000 times, the *p*-value was the proportion when the random *BFLn* was larger than the real value. Genes with p<0.05 were selected as differentially expressed genes (DE-genes).

#### c. Identification of cancer-risk modules

The online module mining tool GraphWeb (http://biit.cs.ut.ee/graphweb/) [48] was chosen to find co-expression modules. GraphWeb is designed to analyze individual or multiple merged networks, search for conserved features across multiple species, mine large biological networks for smaller modules, and compare results of high-throughput datasets. Markov Cluster (MCL) [49] algorithm via the GraphWeb tool was applied to prune the network and to find gene modules. The MCL algorithm simulates a stochastic flow in the expression graph and removes edges that are visited infrequently, resulting in a collection of densely connected groups of genes. The parameter of Markov clustering parameter was set to a default value 1.8.

**Figure 2:**
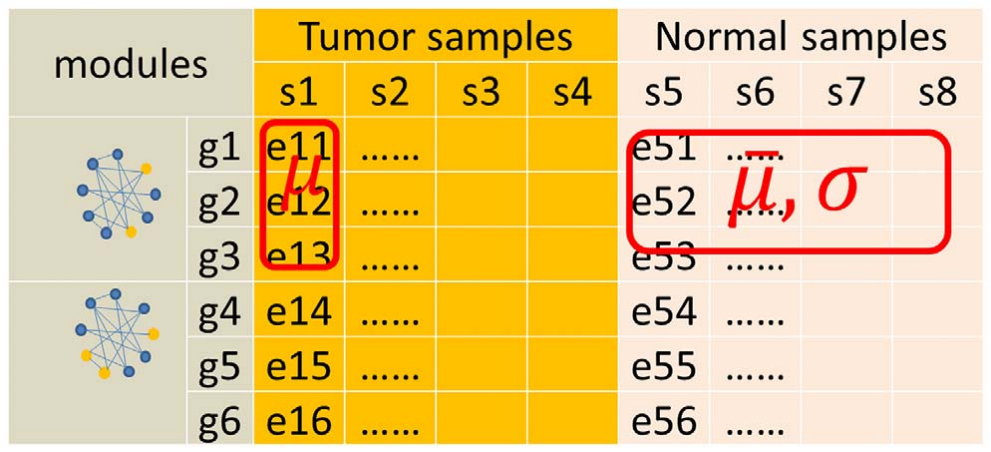
Z-test. Where *μ* means the average expression value of all genes in module1 for the tumor sample s1; e11 is the expression value of g1 in module1 for s1, so do others; 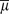 means the average expression value of all genes for all normal samples; σ is the standard deviation of all normal samples.

The candidate modules containing the DE-genes were selected to evaluate the disease risks. Next, *Z*-test[50] was applied to assess the relationship between individual tumor samples and modules (Figure 2).



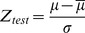



Finally, the significant samples with Z-test higher than the significance threshold (α = 0.05) were picked out. To measure the risk of each module, we defined: 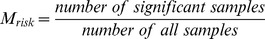



*M_risk_* could be used to assess the disease risk of a candidate module. For each candidate module, 10,000 random modules were constructed by randomly selecting genes from the background gene set with equal numbers of module genes. Then, *M_risk_* was calculated for each random module, and the proportion of modules with *M_risk_* larger than the real value (the significance *p*-value) was computed. Modules with p<0.05 were considered as cancer-risk modules.

#### d. Evaluation of the sample's disease risk

To evaluate the module-based disease risk of each sample, we defined:
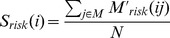





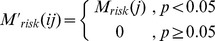



where M includes all cancer-risk modules, *N* is the number of cancer-risk modules, 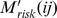 means the cancer-risk of the sample *i* about the module *j*, and *p* is the significance of Z-test.

Cancer-risk modules were applied to evaluate samples by calculating the module-based disease risk of each sample. Then evaluation performance was estimated by a receiver operating characteristic (ROC) curve.

## Results

### The highly-confident co-expression network

**Figure 3:**
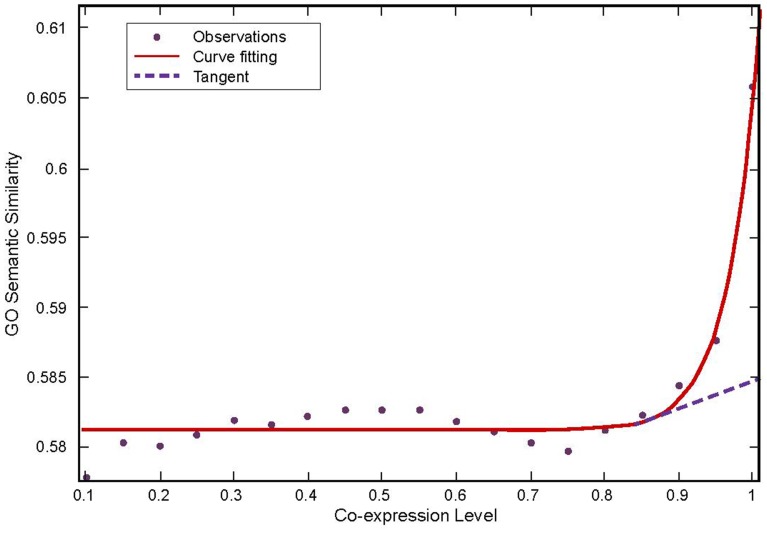
Co-expression Level and GO Semantic Similarity. Purple point means observations, red line indicates the curve fitting, the dotted curve represents the first order tangent.

The Pearson correlation coefficient and the GO semantic similarity of every pair of genes in the expression profile GSE7670 were calculated. After that, curve fitting was applied to analyze the variation trend of average distribution of co-expression value with GO semantic similarity at a 0.05 interval (Figure 3). Functional similarity increased when co-expression level was over the tangency point. Therefore, the pairs of genes with functional similarity over 0.582 and Pearson correlation coefficient over 0.82 (the tangent point) were selected to create the highly-confident co-expression network, which consisted of 9841 nodes and 112,605 edges.

### Cancer-risk Modules

A total of 472 DE-genes were screened out by applying BFLn to the expression profile GSE7670. Then 75 candidate disease modules containing DE-genes were obtained through GraphWeb. After the randomized test, 31 lung cancer-risk modules were obtained (Table 3).

**Table 3:** Lung cancer-risk modules.

Risk	ID	Size	Genes	M_risk_	p-value
high	M2	171	ZEB1, CAV1, HYAL2, MMP12, CLU, TIMP3, DKK3, LPL, TCF21, FOXF1…	1	0.0043
	M72	9	ASPM*,BUB1B,CCNB2,CEP55,KPNA2*,MAD2L1,PBK,TPX2,TRIP13	1	0.0036
	M46	13	BARD1,CDT1,DLGAP5*,DONSON*,GINS1,KIF4A*,	1	0.0062
			MCM7*,MCM3,MLF1IP*,NDC80,PAQR4,TMEM48,TTK		
	M39	14	ADRM1,BYSL,CKS1B,CRABP2,DNAJA3,HAX1,LSM12,	1	0.0067
			MPZL1*,MRPL17,MRPS7,NME4,RPN2,SLC2A4RG,STRA13		
	M281	3	CRYAB*,HSPB2*,VGLL3*	1	0.0018
	M82	9	ALG3*,EIF2S1,HSPB11,LRRC42,MCTS1,P4HA2,PSMA5,SEC61G,VARS	1	0.004
	M61	11	ADAMTS8*,CSRP1*,KCNK3*,LINC00312*,MYH11*,	1	0.0058
			MYLK,PDE2A,PKNOX2*,RASL12*,SETBP1,TACC1*		
	M266	3	CDCA3,GALNT6,IDH2*	1	0.0017
	M340	3	MRPS34,NUBP2,SNRNP25*	1	0.0015
	M363	3	DDR1*,FLAD1*,SPINT1	1	0.002
middle	M62	11	CCNB1,CKAP2,KIF11*,KIF20A*,MCM4,MELK*,	0.9642	0.0187
			NCAPG,NETO2*,PRC1*,SHCBP1,TOP2A*		
	M27	17	CCT6A*,EIF2AK1*,EIF3B,FKBP14*,GART,GINS4,GNL3,	0.9642	0.0304
			HEATR2*,KLHL7*,LSM5*,MRPS17*,MRPS33*,PHLDA2,		
			POLD2,PPP1R14B*,PSMD2,TMEM106B*		
	M268	3	HPRT1*,SCRN1*,TPBG*	0.9642	0.0065
	M102	8	AVL9,CDK5,CORO1B,CHPF2*,ITPKA,NDUFS8,PPP1CA,SSH3	0.9642	0.0172
	M63	10	A2M*,CASP1*,CD97*,FABP4*,GAS6*,GMFG*,	0.9642	0.0171
			PDLIM2*,PLEKHO2*,RARRES2,TRPV2*		
	M54	12	CLDN5*,CRIM1*,DOCK6,FGR*,ICAM2*,INPP1*,	0.9642	0.0223
			KANK3*,LIMS2*,LRRC32,PCDH12*,PTGIR*,RASIP1*		
	M258	4	FZR1*,CLDN4,LY6E,PRSS8	0.9642	0.0076
	M188	5	BLVRA,KIAA0391,PSMA6,SRP54*,TFPI2	0.9642	0.0091
	M297	3	AHCY*,PKP3,SLC38A1	0.9642	0.0088
	M321	3	GLO1,EGFL7,PDXDC1*	0.9642	0.0056
	M180	5	DHTKD1*,MEA1,SLC35A2,TMED3*,TPMT	0.9642	0.0096
	M86	9	CDKL2,ENY2,HAND1,LY6D,ORM1,ORM2,RAB25*,S100G,TSTA3*	0.9642	0.0159
	M387	3	GALNTL2*,SAR1B*,TSPAN6*	0.9642	0.0062
low	M157	5	DHFR,DTL,GMPS,MYBL2,RFC4*	0.9285	0.0234
	M241	4	COG8,FAM158A,PDF,PSMB5*	0.9285	0.0207
	M249	4	KRT10,NIPSNAP1*,POLDIP2*,SEPHS2	0.9285	0.0173
	M314	3	FAM65A*,GIMAP5*,SEPP1*	0.9285	0.0159
	M280	3	GYPC,PTGDS*,RPL15	0.9285	0.016
	M144	6	BCKDK,DECR2,GALE,NDUFB11,PYCR1*,RRNAD1	0.9285	0.028
	M316	3	CTSA,ERGIC3,PAFAH1B3*	0.8928	0.0313

Risk is modules category, ID indicate the identifier of cancer-risk modules, size is the module scale, namely the number of genes in the module, genes is the genes in the modules and the genes which were marked * were DE-genes, M_risk_ is the cancer risk of modules, p-value is significance p value of random randomized test.

### Evaluation of cancer-risk modules

**Figure 4:**
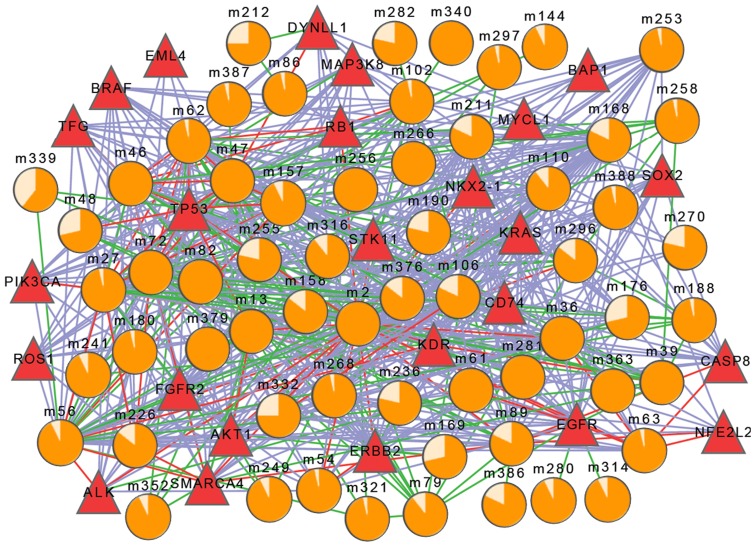
The relationship network of cancer-risk modules and lung cancer genes. The circles indicate cancer-risk modules, and the proportion of orange parts indicates cancer risk (*M_risk_*). The disease-causing genes is represented by red triangles. Edges' colors indicate the relationships, purple represents for the protein-protein interaction, green for function sharing, and red for both functional and interaction relationship.

The cancer-risk modules were evaluated at the functional level and interactional level. On one hand, functional enrichment was performed for each lung cancer-risk module using an online tool DAVID (http://david.abcc.ncifcrf.gov/home.jsp) [51], and then significantly enriched GO terms of each module were obtained (More modules are in the Table S1). On the other hand, the interactional relationships of modules were assessed using protein interaction data from iRefWeb. The relationship network of cancer-risk modules and known lung cancer genes was constructed on the basis of functional and interactional relationships (Figure 4). The results showed that lung cancer-risk modules were closely related with the lung cancer genes, which indicated that these modules and genes might synergistically cause lung cancer. For instance, m46 was associated with cell cycle regulation and phosphorylation [52], cell proliferation and cell cycle checkpoint [53], and ATP binding [54] by interacting with known lung cancer genes KRAS, KDR and TP53, respectively. These functions were confirmed to be related to the occurrence of lung cancer. Another module m63 was significantly enriched in functions associated with the cancer, e.g. the response to corticosteroid stimulus, the response to organic substance, and glucocorticoid stimulus and steroid hormone stimulus together by interacting with known lung cancer genes KRAS, NFE2L2 and NKX2, respectively [55], [56], [57].

To further analyze the relationship network, the cancer-risk modules were classified into three types according to the risks: the high, middle and low risk modules (Table 3), and the corresponding degree distributions were calculated (Table 4). The results showed that the high risk modules tend to have high degrees. Namely, they had more connections with other modules and known disease genes at the functional and interactional levels. They played pivotal roles in the network.

**Table 4:** Average Degree for three types of cancer-risk modules.

Risk	D_W	D_M	D_D	D_P	D_F	D_B
High	13.00	5.222	7.78	6.33	9.22	2.50
Middle	10.67	3.75	6.92	3.50	8.58	1.42
Low	4.71	2.00	2.70	2.14	2.80	0.28

D_W stands for degree of whole net, D_M for degree only between modules, D_D for degree only considered of modules with disease-causing genes, D_P for degree of the protein interaction edges(purple edges), D_F for degree of function edges(green edges), D_B for degree of both protein interaction and function(red edges).

### Evaluation of the module-based disease risk

**Figure 5:**
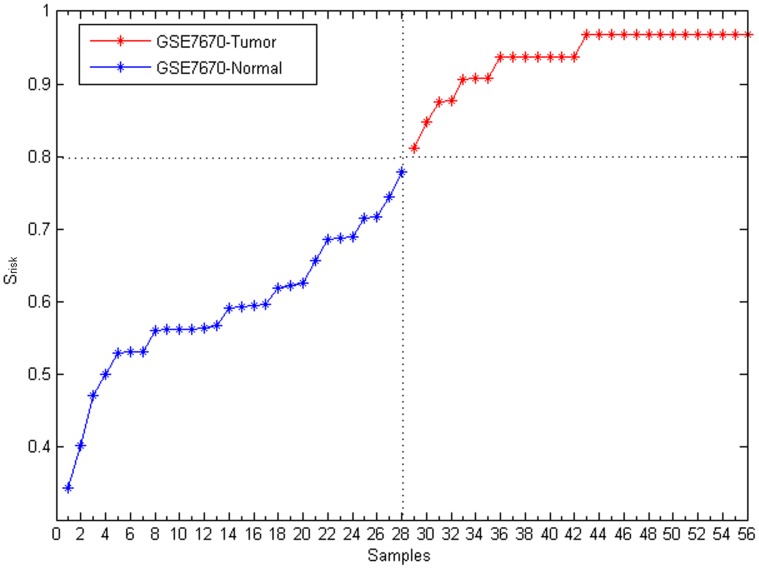
The lung cancer risk of each sample in GSE7670. X-axis is samples. Y-axis is the lung cancer risk score of individual samples, and it is ranked from smallest to largest. Red represents lung cancer samples; and blue represents normal samples.

The lung cancer risk of each sample was evaluated by considering the cancer-risk modules. By measuring the lung cancer risk (*S_risk_*), every sample in GSE7670 was evaluated. It turned out that every sample could be successfully identified as disease (*S_risk_*>0.8) or normal (*S_risk_*<0.8) based on its disease risk (Figure 5).

### The robustness of our method

**Figure 6:**
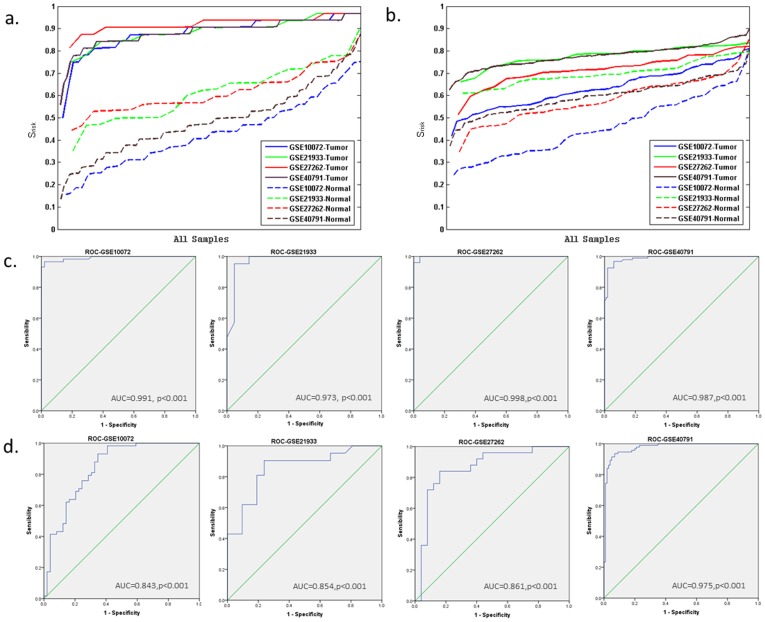
The robustness of our method and comparison with the WGCNA method. **a**) X-axis is samples. Y-axis is the lung cancer risk score of individual samples using our method, and it is ranked from the smallest to the largest. Blue represents GSE10072; green represents GSE21933; red represents GSE27262; and brown represents GSE4079. Full lines represent lung cancer samples; and dashed lines represent normal samples. The different experiment data sets have different numbers of the normal samples and the disease samples. In order to show the disease risk of every sample in four expression profiles intuitively, all samples of each expression profiles are distributed uniformly throughout x-axis. **b**) The figure is plotted the same way as a). The lung cancer risk of each sample is evaluated by the WGCNA method. **c**) Receiver operator characteristic curve using our method for the four lung cancer expression profiles (see Figure 7a). The areas under curve provided at lower right of each diagram. **d**) Receiver operator characteristic curve using the WGCNA method for the four lung cancer expression profiles (see Figure 7b).

In order to verify the robustness of this method, first, other four expression profiles (GSE10072 from GPL96, the same as GSE7670; GSE27262 and GSE40791 from GPL570; and GSE21933 from GPL6254) about lung cancer and normal were evaluated, respectively (Table 1). The results showed that the module-based disease risks of cancer samples were higher than those of normal ones (Figure 6a). ROC curves were then plotted and the AUC values (>0.97) were used to measure the evaluation performances of the cancer-risk modules which were obtained by our method (Figure 6c). The method had good performance in the expression profiles not only from the same platform, but also from different platforms.

**Figure 7:**
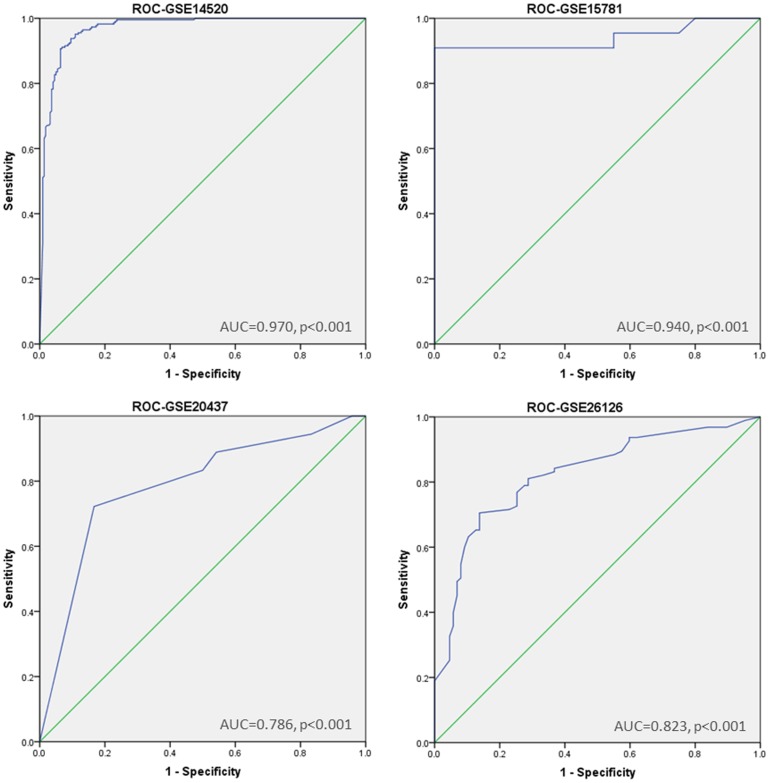
Receiver operator characteristic curve for expression profiles of liver cancer (GSE14520), colon cancer (GSE15781), breast cancer (GSE20437), and prostate cancer (GSE26126).

Next, we identified risk modules of liver cancer (GSE14520), colon cancer (GSE15781), breast cancer (GSE20437), and prostate cancer (GSE26126) in the same way, respectively (More cancer-risk modules information in the four cancers are in the Table S3). The cancer-risk modules were used to evaluate the disease risks of the samples, and the corresponding ROC curves were drawn (Figure 7).

### Method comparisons

**Figure 8:**
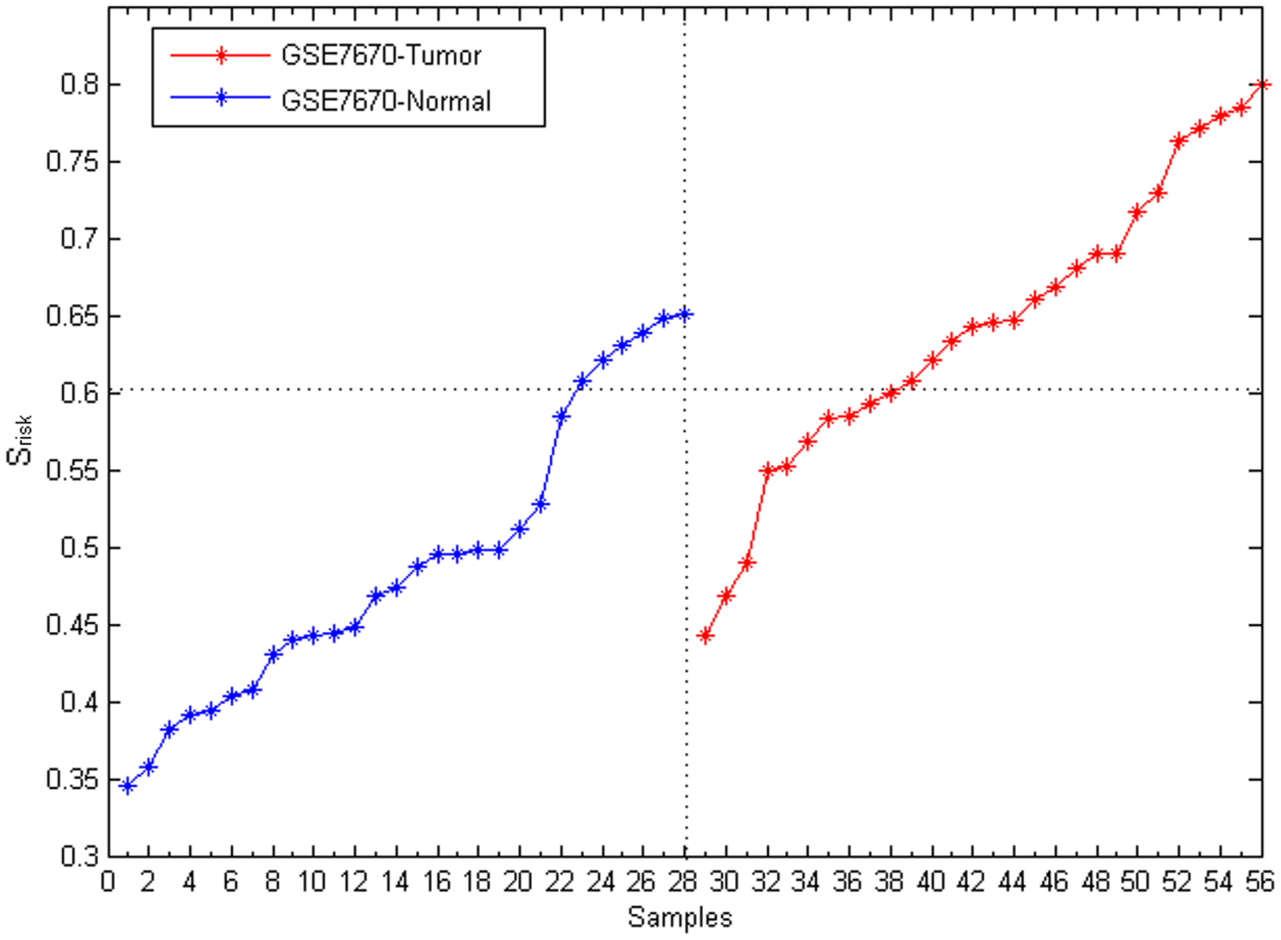
The lung cancer risk of each sample in GSE7670 by the WGCNA method.

The WGCNA method [24] is a widely used technique to construct gene modules within a network based on gene co-expression relationships. In this paper, the accuracy and robustness of WGCNA and our method were compared. Fifty seven lung cancer risk modules were obtained from GSE7670 using the WGCNA method. The lung cancer risk of every sample in GSE7670 itself was evaluated with the modules. Cancer risks of some cancer samples were smaller than those of normal ones (Figure 8), which indicated the WGCNA method could not completely identified samples as disease or normal as accurately, while our method could (Figure 5).

Then the evaluation of the samples' lung cancer risks was extended to other four expression profiles about lung cancer and normal (Figure 6b). It was found that the cancer risks of cancer samples were not significantly different from those of normal ones. The ROC curves were then used to evaluate the performance of the WGCNA method (Figure 6d). We found that our method had better accuracy and robustness than the WGCNA method (Figure 6).

## Discussion

Studying the mechanisms of diseases by analyzing gene expression profiles appears to be a convenient and effective way. Considering the functional similarity could better reflect the function information of the disease. In this paper, a new method was proposed to identify thirty one cancer-risk modules and evaluate the module-based disease risks of samples by using a co-expression network with functional similarity information. Finally, the relationship network of cancer-risk modules and cancer genes was constructed on the functional level and interactional level.

These modules were found to be closely related to cancers in the aspects of functions, interactions, and literature. Our method was proved to be fairly robust by evaluating the disease risks of samples in four lung cancer expression profiles and in four other cancers, and had better performance than the WGCNA method.

Cancer-risk modules and the evaluation of the module-based disease risk from this study were confirmed to be credible with the following considerations. (i) Differentially expressed genes were selected by using the BFLn method, which considered both gene expression and sample label distribution so as to eliminate outliers caused by bias expression of individual gene or experiment errors. (ii) Our gene network was of high confidence, because the method was used to calculate not only the co-expression correlation, but also functional similarities between genes. The gene pairs with both high expression consistency and functional similarity were retained for building the high confident network, which was capable of avoiding biased results merely depending on expression. (iii) The cancer risks of modules were evaluated by using the proportion of significant tumor samples, which could be a new method to evaluate disease modules. The genes in cancer-risk modules could be potential disease genes, and might act as drug targets for the treatment of aggressive cancers. All genes of m46 were related with lung cancer. For instance, MCM7 is a significant subunit of MCM complex, which could be a novel therapeutic target in lung cancer [58]. Another gene BARD1, whose isoforms may be related to tumor initiation and invasive progression, was a more suitable neoteric prognostic marker for non-small-cell lung cancer [59]. KIF4A might hold a promise for the development of anticancer drugs and cancer vaccines as well as a prognostic biomarker in clinic [60]. For the genes in module m63, A2M was in limited and extended lung cancer patients compared to a nonsmoker and smoker control population [61], FABP4 was down-regulated in lung adenocarcinoma [62], and CASP1 affected the single-nucleotide polymorphisms, increasing the cancers risk [63]. (iv) The evaluation of samples' module-based disease risks is accuracy and robustness. Because our method integrated the differentially expressed genes, a co-expression network and functional similarities, the cancer-risk modules were closely related to the pathogenesis of cancer in the aspects of functions and interactions. On the functional level, the cancer-risk modules could reflect the functional classes related to diseases; on the interactional level, the cancer-risk modules could be very high correlated with the disease genes.

**Figure 9:**
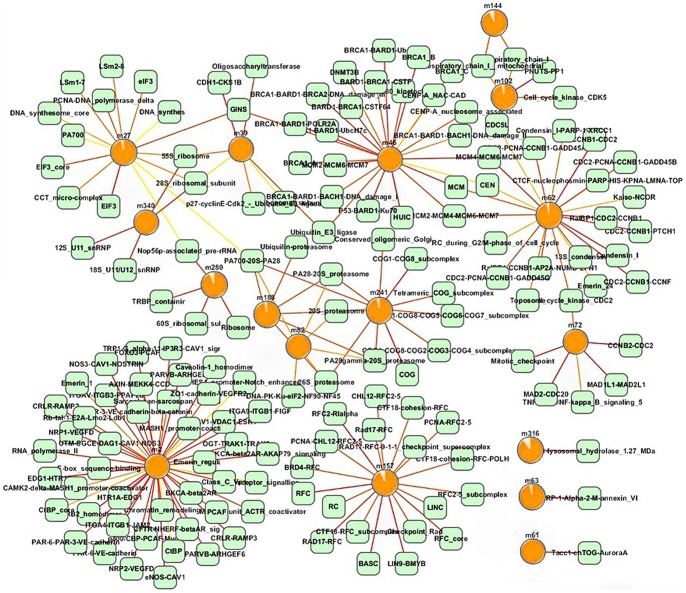
Overlapping relationship network of cancer-risk modules and complexes. The circles indicate cancer-risk modules, and the proportion of orange parts indicates cancer risk (*M_risk_*). The green squares indicate complexes. Edges indicate cancer-risk modules and complexes sharing at least one gene. The more the number of shared genes are, the redder the edges are.

Additionally, we investigated the overlap between the cancer-risk modules and the protein compounds (Figure 9). The results of hypergeometric distribution analysis showed that 17 modules had significant overlap with 150 complexes (p<0.05). For example, module m46 shared genes with 24 complexes, among which 19 complexes had an overlap rate higher than 20%. The complex BRCA1_A recruited BRCA1 to DNA damage sites [64]. Partial depletion of Mcm proteins which were typically loaded in excessive number of locations led to cancers and stem cell deficiencies [65]. The expression of ubiquitin E3 ligase was associated with estrogen receptor (ER)-positive status in human breast tumors [66] (More modules and complex information are in the table S2). Our method will be more comprehensive considering protein-protein information to construct an integrated network and developing a module mining algorithm in the future.

In conclusion, this study presented a novel method to evaluate disease risks of samples based on cancer-risk modules and to analyze the relationships between the disease and modules. This method could provide assistance to the diagnosis and treatment of cancers and a new clue for revealing the cancer mechanisms.

## Supporting Information

Table S1 **The GO information of cancer-risk modules.**
(DOC)

Table S2 **Cancer-risk modules and complexs.**
(DOC)

Table S3 **The cancer-risk modules in the other four cancers.**
(DOC)
