# Supplementary material for: Cancer-Risk Module Identification and Module-Based Disease Risk Evaluation: A Case Study on Lung Cancer
Source: PLoS One. 2014 Mar 18;9(3):e92395. doi: 10.1371/journal.pone.0092395 (PMC3958511; doi:10.1371/journal.pone.0092395)
Supplement: Table S1 — The GO information of cancer-risk modules. (DOC) [file pone.0092395.s001.doc]

Table S1 The GO information of cancer-risk modules

| ID | Score | Size | Genes | function_description (p<0.05) | gene_description |
| --- | --- | --- | --- | --- | --- |
| M2 | 1 | 171 | ZABCA8*,ACVRL1*,ADAMTSL3*,ADH1B*,ADRB2*,AGER*,ANGPT1*,ANKS1A*,AOC3*,ARHGEF10*,ARHGEF3*,ARHGEF6,ARMCX1,ASPA*,BCHE*,BTBD3*,C6ORF1,CAV1*,CBX7*,CCDC48*,CCDC68*,CD36,CD93*,CDH5*,CES1*,CHRDL1*,CLEC3B*,CLU,COX7A1*,DENND3*,DIXDC1*,DKK2,DKK3*,DPT*,DPYSL2*,EDNRB*,EFEMP1*,EML1*,EMP2*,FAM107A*,FAM125B*,FAM134B,FAM189A2,FAM190B,FBXL5*,FCN3*,FGFR4*,FIGF*,FMO3*,FOXF1*,FOXF2*,FXYD1*,FXYD6*,FYCO1,GABARAPL1,GABARAPL3,GATA6*,GDF10*,GFOD1*,GHR*,GIMAP6*,GNG11*,GPC3*,GPM6A*,GPM6B*,GPR126,GPX3*,GRK5*,H2AFJ*,HEG1*,HIGD1B*,HPCAL1*,HSPB8*,HYAL2*,IL33*,INPP5A*,INPP5K*,ITIH5*,ITM2A*,JAM2*,JAM3,KAL1*,KAT2B*,KIAA0355,KIAA1462*,KL*,KLF11*,KLF2*,LDB2*,LHFP*,LIMCH1*,LINC00341,LMCD1,LMO2*,LMO7*,LPHN2*,LPL*,LRRN3*,MAOB*,MAP4,MAPRE2,MEIS1*,MEIS2*,MFAP4*,MMP12,MMRN2*,MOGAT3,MYL9*,NDNF,NDRG4*,NEDD4L*,NPR3,OLFML1*,OR7E47P*,P2RY14*,PALM2*,PALM2-AKAP2*,PALMD*,PAPSS2*,PDE3B,PHACTR2,PIKFYVE,PLSCR4*,PODXL*,PPAP2B*,PRKCH*,PTGER4*,PTPRB*,PTPRD*,PTPRM,PXMP2,RAI2,RAMP2*,RAMP3*,RFTN1*,RGS5,S1PR1*,SEC14L1*,SEMA3G*,SEMA5A*,SEPP1,SESN1*,SGCG*,SH3BP5*,SLC6A4*,SLIT2,SNRK*,SPARCL1*,SPOCK2*,SRPX*,STX7*,SYNPO*,TBX3*,TCF21*,TGFBR3*,TIE1*,TIMP3*,TIRAP,TMEM204,TMEM47*,TMOD1*,TRAK2*,TSC22D1,TSPAN7*,UBL3*,WASF3*,WIF1*,ZBTB16,ZEB1,ZFP106*,ZNF423, | GO-0007155~cell_adhesion,  GO-0045934~negative_regulation_of_nucleobase,_nucleoside,_nucleotide_and_nucleic_acid_metabolic_process,  GO-0045935~positive_regulation_of_nucleobase,_nucleoside,_nucleotide_and_nucleic_acid_metabolic_process  GO-0007568~aging,  GO-0042060~wound_healing,  GO-0019838~growth_factor_binding,  GO-0042127~regulation_of_cell_proliferation,  GO-0001525~angiogenesis,  GO-0001525~angiogenesis,  GO-0030324~lung_development,  GO-0016791~phosphatase_activity,  GO-0008015~blood_circulation,  GO-0003779~actin_binding,  GO-0006898~receptor-mediated_endocytosis,  GO-0007411~axon_guidance,  GO-0007187~G-protein_signaling,_coupled_to_cyclic_nucleotide_second_messenger,  GO-0007188~G-protein_signaling,_coupled_to_cAMP_nucleotide_second_messenger,  GO-0060425~lung_morphogenesis,  GO-0048598~embryonic_morphogenesis,  GO-0030334~regulation_of_cell_migration,  GO-0030155~regulation_of_cell_adhesion,  GO-0019899~enzyme_binding,  GO-0048514~blood_vessel_morphogenesis  GO-0030099~myeloid_cell_differentiation,  GO-0001568~blood_vessel_development,  GO-0045941~positive_regulation_of_transcription,  GO-0019933~cAMP-mediated_signaling,  GO-0042577~lipid_phosphatase_activity,  GO-0016044~membrane_organization,  GO-0007242~intracellular_signaling_cascade,  GO-0006928~cell_motion | We sought to identify the subset of genes regulated in lung cancer by ZEB1, an E-box transcriptional repressor known to induce. EMTPMID: 20980099  These results indicated that LKB1 inactivation triggers EMT in lung cancer cells through the induction of ZEB1. PMID: 20207041  These results show an inverse relationship between E-cadherin and ZEB1 and a direct relationship between COX-2 and ZEB1 by immunohistochemical staining of human lung adenocarcinoma tissue sections. PMID: 16707460  Among known transcriptional suppressors  of E-cadherin, ZEB1 was uniquely correlated with E-cadherin loss in lung cancer cell lines, and its inhibition by RNA interference resulted in E-cadherin induction. PMID: 12937339  CAV1 expression was tightly linked to the ability to grow attached to the plastic cell culture surface, whereas CAV1-nonexpressing lung cancers of both SCLC and NSCLC type grew as suspension cultures. PMID: 15205342  In this model, MMP12-Flag fusion protein overexpression and its increased enzymatic activity were observed in the lung in an inducible manner, which led to inflammatory cell infiltration and increased epithelial growth. PMID: 19706765  These results indicated lower metastatic potential for cancer cells with high CLU level. PMID: 21630085  In vitro, CLU silencing by antisense oligonucleotides (ASO) and small-interfering  RNAs (siRNA) directed against CLU mRNA in CLU-rich lung cancer cell lines sensitized cells to chemotherapy and radiotherapy and decreased their metastatic potential.  PMID: 19879423  These findings which TIMP-3 gene therapy offers a therapeutic advantage over TIMPs 1 and 2 establish the potential of adenoviral gene delivery of TIMP3 as a therapeutic agent for selected lung cancers. PMID: 16860902  We investigated another function of DKK3 in non-small cell lung cancer H460 cells, in which DKK3 was hypermethylated (44%) but still expressed, by interfering with DKK3 expression using DKK3-silencing RNA (SiRNA). PMID: 20514419  Wo find that the higher LPL activity in lung cancer tissue provides a possible mechanism for increasing the supply of lipid nutrients to the tumor, necessary for tumor growth. PMID: 17620184  Cell lines from other cancer types also showed frequent TCF21 promoter hypermethylation. PMID: 20945327  Wo revealed that FoxF1 regulates the contractility of fibroblasts, their production of hepatocyte growth factor and fibroblast growth factor-2, and their stimulation of lung cancer cell migration through Studies with FoxF1 gain- and loss-of-function fibroblasts. PMID: 20233876 |
| M72 | 1 | 9 | ASPM*,BUB1B,CCNB2,CEP55,KPNA2*,MAD2L1,PBK,TPX2,TRIP13, | GO-0007049~cell_cycle,  GO-0000279~M_phase,  GO-0007067~mitosis,  GO-0005524~ATP_binding,  GO-0008283~cell_proliferation,  GO-0051301~cell_division,  GO-0022403~cell_cycle_phase,  GO-0000166~nucleotide_binding,  GO-0000075~cell_cycle_checkpoint | ASPM were upregulated in immortalized cells, cancer cells, and non-small-cell lung cancer  (NSCLC) tissues. PMID: 19160420  Protein levels of KPNA2 in pleural effusion from NSCLC patients were significantly higher than those from non-lung cancer. Moreover, knockdown of KPNA2 inhibited the migration ability and viability of lung cancer cells. PMID: 20658535  These results suggest that genetic variants in MAD1L1 and MAD2L1 confer susceptibility to lung cancer, which might result from reduced spindle checkpoint function due to attenuated function of MAD1L1 and/or MAD2L1. PMID: 20516147  The present study appraised the significance of TPX2 aberrant expression for tumorigenesis and progression of human squamous cell carcinoma(SCC) in lung. PMID: 16489064 |
| M46 | 1 | 13 | BARD1,CDT1,DLGAP5*,DONSON*,GINS1,KIF4A*,MCM3,MCM7,MLF1IP*,NDC80,PAQR4,TMEM48,TTK, | GO-0007049~cell_cycle,  GO-0000279~M_phase,  GO-0010564~regulation_of_cell_cycle_process,  GO-0006260~DNA_replication,  GO-0005524~ATP_binding,  GO-0008283~cell_proliferation,  GO-0051726~regulation_of_cell_cycle,  GO-0022403~cell_cycle_phase | Wo find the pattern of BARD1 isoform expression  was similar in tumor and morphologically normal peri-tumor tissues, and only one novel isoform π was specifically upregulated in tumors PMID: 21815143  Wo find Mutations in tumor-suppressor gene BARD1 Appear in the inherited and spontaneous breast, ovarian and uterine cancers. PMID: 16152612  Suppression of MCM7 using specific siRNAs inhibited incorporation of BrdU in lung and bladder cancer cells overexpressing MCM7, and suppressed the growth of those cells more efficiently than that of normal cell strains expressing lower levels of MCM7. PMID: 21619671  NEK2(p<0.001) and TTK (p = 0.002) expression in the noninvolved lung tissue was associated with a 3-fold increased risk of mortality from lung adenocarcinoma in smokers. PMID: 18297132 |
| M39 | 1 | 14 | ADRM1,BYSL,CKS1B,CRABP2,DNAJA3,HAX1,LSM12,MPZL1*,MRPL17,MRPS7,NME4,RPN2,SLC2A4RG,STRA13, |  | Aberrant methylation was detected in four genes (CRABP2, NOEY2, T,MAP2K3) in at least one lung adenocarcinoma cell lines. PMID: 17980147  The results reveal statistically important HAX1 up-regulation in breast cancer, lung cancer and melanoma, along with some minor variations in the splicing pattern. PMID: 20196840 |
| M281 | 1 | 3 | CRYAB*,HSPB2*,VGLL3* |  | - |
| M82 | 1 | 9 | ALG3*,EIF2S1,HSPB11,LRRC42,MCTS1,P4HA2,PSMA5,SEC61G,VARS |  | - |
| M61 | 1 | 11 | ADAMTS8*,CSRP1*,KCNK3*,LINC00312*,MYH11*,MYLK,PDE2A,PKNOX2*,RASL12*,SETBP1,TACC1* | GO-0003779~actin_binding | - |
| M266 | 1 | 3 | CDCA3,GALNT6,IDH2* |  | - |
| M379 | 1 | 3 | ARHGEF16,MED20*,PSAT1, |  | - |
| M340 | 1 | 3 | MRPS34,NUBP2,SNRNP25* |  | - |
| M363 | 1 | 3 | DDR1*,FLAD1*,SPINT1 |  | Wo find high DDR1 levels in human lung tumors were associated with poor survival.PMID: 22223527  It is now clear that aberrant signaling through the DDR1 receptor is closely associated with various steps of tumorigenesis. PMID: 21398698  These findings indicate that up-regulation of DDR1 may contribute to the progression and poor prognosis of NSCLC and this effect may be associated with increased invasiveness. PMID: 20596615 |
| M62 | 0.9642 | 11 | CCNB1,CKAP2,KIF11*,KIF20A*,MCM4,MELK*,NCAPG,NETO2*,PRC1*,SHCBP1,TOP2A* | GO-0007049~cell_cycle,  GO-0000279~M_phase,  GO-0007067~mitosis,  GO-0005524~ATP_binding,  GO-0051301~cell_division,  GO-0003774~motor_activity,  GO-0022403~cell_cycle_phase,  GO-0000166~nucleotide_binding,  GO-0007059~chromosome_segregation | In tumour samples, as compared to normal lung tissue, the up-regulated genes included such known tumour markers as CCNB1, PLK, tenascin, KRT8, KRT19 and TOP2A. PMID: 12173052  We determined the relevance of MCM4 in proliferation of NSCLC by downregulating its expression with small-interfering RNA in three NSCLC cell lines. PMID: 20884074  Primary tumors with inactivation of both FHIT and p53 displayed the strongest deregulation of growth-related pathways with high levels of expression of CCNB1, BUB1, CDC6, TOP2A, MCM6, and CENPF. PMID: 22425911  The data presented herein suggest that the expression of HER2 did not influence the SM-induced apoptosis of different types of lung cancer cells and that the SM up-regulation of HER2 and TOP2A expressions simultaneously augmented trastuzumab and epirubicin-induced deaths of lung cancer H661 and H69 cells. PMID: 18078328 |
| M27 | 0.9642 | 17 | CCT6A*,EIF2AK1*,EIF3B,FKBP14*,GART,GINS4,GNL3,HEATR2*,KLHL7*,LSM5*,MRPS17*,MRPS33*,PHLDA2,POLD2,PPP1R14B*,PSMD2,TMEM106B* | GO-0006412~translation,  GO-0006412~translation | We previously identified PSMD2, a subunit of the 19S regulatory complex of proteasomes, as a constituent of a signature associated with the acquisition of metastatic phenotype and poor prognosis in lung cancers.  PMID: 21465578 |
| M268 | 0.9642 | 3 | HPRT1*,SCRN1*,TPBG* |  | The least variance and linear regression analysis demonstrated that GAPDH and HPRT had the strongest correlation in pooled tumour and normal lung tissues PMID: 16319328 |
| M102 | 0.9642 | 8 | AVL9,CDK5,CORO1B,CHPF2*,ITPKA,NDUFS8,PPP1CA,SSH3 | GO-0016311~dephosphorylation,  GO-0051301~cell_division | A recent study detected overexpression of the CDK5 gene in non-small-cell lung cancer. PMID: 19343042  Multiple downstream components of the EGFR-family-signaling pathway, including CDK5, AKT1 and SHC1, are overexpressed as a direct result of gene amplification in lung cancer. PMID: 18391978  We find that the observed expression of ITPKA early in tumor development increases the metastatic potential of lung adenocarcinoma cells. PMID: 21792881 |
| M63 | 0.9642 | 10 | A2M*,CASP1*,CD97*,FABP4*,GAS6*,GMFG*,PDLIM2*,PLEKHO2*,RARRES2,TRPV2* |  | The results showed that A2M and A1PI were increased in the involved lung from limited cancer when compared to normals. PMID: 2476329  Wo find Axl expression appears to be a consequence of cellular adhesion and possibly influences differentiation in human lung cancers. PMID: 11677117 |
| M54 | 0.9642 | 12 | CLDN5*,CRIM1*,DOCK6,FGR*,ICAM2*,INPP1*,KANK3*,LIMS2*,LRRC32,PCDH12*,PTGIR*,RASIP1* | GO-0007155~cell_adhesion  GO-0016337~cell-cell_adhesion | - |
| M258 | 0.9642 | 4 | FZR1*,CLDN4,LY6E,PRSS8 |  | - |
| M188 | 0.9642 | 5 | BLVRA,KIAA0391,PSMA6,SRP54*,TFPI2 |  | The expression level of the proteins in lung cancer patient sera was assayed by an immunoaffinity-multiple reaction monitoring method, and the results were comparable with those obtained from ELISA. PMID: 18388126 |
| M297 | 0.9642 | 3 | AHCY*,PKP3,SLC38A1 |  | A high level of PKP3 expression was associated with poor survival as well as disease stage and node status for patients with lung adenocarcinoma, suggesting an important role of the protein in development and progression of this disease 。PMID: 16103059 |
| M321 | 0.9642 | 3 | GLO1,EGFL7,PDXDC1* |  | As a result, our study demonstrates that miR-126 can inhibit proliferation of non-small cell lung cancer cells through one of its targets, EGFL7. PMID: 20034472  Positive correlation between cellular GLO1 activity and BBGC sensitivity was observed in the lung cancer cell lines.PMID: 11489834 |
| M180 | 0.9642 | 5 | DHTKD1*,MEA1,SLC35A2,TMED3*,TPMT |  | - |
| M86 | 0.9642 | 9 | CDKL2,ENY2,HAND1,LY6D,ORM1,ORM2,RAB25*,S100G,TSTA3* | -GO-0006953~acute-phase_response | Of the 64 genes, 11 are related to cancer metastasis (APC, CDH8, IL8RB, LY6D, PCDHGA12, DSP, NID, ENPP2, CCR2, CASP8, and CASP10) and eight are involved in apoptosis (CASP8, CASP10, PIK3R1, BCL2, SON, INHA, PSEN1, and BIK). PMID: 17194181  Eight (TGFBR3, RUNX3, TRGC2, TRGV9, TARP, ACP1, VCAN, and TSTA3)differentiated paired tumor versus noninvolved lung tissue samples in stage I cases, suggesting a similar pattern of lung cancer-related changes in PWB and lung tissue. PMID: 21742797 |
| M387 | 0.9642 | 3 | GALNTL2*,SAR1B*,TSPAN6* |  | - |
| M157 | 0.9285 | 5 | DHFR,DTL,GMPS,MYBL2,RFC4* | GO-0006260~DNA_replication  GO-0000166~nucleotide_binding  GO-0034404~nucleobase,_nucleoside_and_nucleotide_biosynthetic_process | The associated increase in DHFR expression resulted in increased resistance to methotrexate but had no effect on other classes of anticancer agents. PMID: 8797766  Meta-analysis of gene expression data sets from lung squamous cell, breast, colon, prostate, and pancreas carcinomas, as well as glioblastoma, revealed that a subset of PKCiota target genes, particularly COPB2 and RFC4, correlate with PKCiota expression in many tumor types. PMID: 19223491 |
| M241 | 0.9285 | 4 | COG8,FAM158A,PDF,PSMB5* |  | The confirmation of PDF reproducibility over times much shorter than stereotactic body radiotherapy delivery duration is a vital part of the initial validation process of probability-based treatment planning for stereotactic body radiotherapy for lung cancer. PMID: 18954717 |
| M249 | 0.9285 | 4 | KRT10,NIPSNAP1*,POLDIP2*,SEPHS2 |  | - |
| M314 | 0.9285 | 3 | FAM65A*,GIMAP5*,SEPP1* |  | We found significant down-regulation of SEPP1 expression level in tumorous lung tissue (2.732-fold; p<0.001). The expression of hGPX1 and SEP15 in tumorous tissue remained unchanged compared to healthy tissue. PMID: 19058871 |
| M280 | 0.9285 | 3 | GYPC,PTGDS*,RPL15 |  | A comprehensive statistical analysis suggested the set of 19 gene markers, ANKRD28, BHLHE40, CGGBP1, RBSP3, EPHB1, FGD5, FOXP1, GORASP1/TTC21, IQSEC1, ITGA9, LOC285375, LRRC3B, LRRN1, MITF, NKIRAS1/RPL15, TRH, UBE2E2, VHL, WNT7A, to allow early detection, tumor progression, metastases and to discriminate between SCC and ADC with sensitivity and specificity of 80-100%. PMID: 22491060 |
| M144 | 0.9285 | 6 | BCKDK,DECR2,GALE,NDUFB11,PYCR1*,RRNAD1 | GO-0055114~oxidation_reduction | - |
| M316 | 0.8928 | 3 | CTSA,ERGIC3,PAFAH1B3* |  | - |

Notes: function_description selected the enriched GO terms with significance p<0.05.
