# Supplementary material for: Cancer-Risk Module Identification and Module-Based Disease Risk Evaluation: A Case Study on Lung Cancer
Source: PLoS One. 2014 Mar 18;9(3):e92395. doi: 10.1371/journal.pone.0092395 (PMC3958511; doi:10.1371/journal.pone.0092395)
Supplement: Table S2 — Cancer-risk modules and complexs. (DOC) [file pone.0092395.s002.doc]

Table S2 cancer-risk modules and complex information

| Modules | Complexs |
| --- | --- |
| m102 | CDC5L  Respiratory_chain_I__mitochondrial  Respiratory_chain_I  Cell_cycle_kinase_CDK5  PNUTS-PP1 |
| m144 | Respiratory_chain_I__mitochondrial  Respiratory_chain_I |
| m157 | RC  LINC  Checkpoint_Rad  RAD17-RFC-9-1-1_checkpoint_supercomplex  CTF18-cohesion-RFC-POLH  CTF18-cohesion-RFC  BRD4-RFC  PCNA-CHL12-RFC2-5  Rad17-RFC  CHL12-RFC2-5  CTF18-RFC_subcomplex  PCNA-RFC2-5  RFC2-5_subcomplex  RFC_core  RFC2-RIalpha  LIN9-BMYB  RAD17-RFC  RFC  BASC |
| m188 | PA700-20S-PA28  26S_proteasome  PA28-20S_proteasome  PA28gamma-20S_proteasome  20S_proteasome  Ubiquilin-proteasome |
| m2 | CtBP  HES1_promoter-Notch_enhancer  P2X7_receptor_signalling  CtBP_core  Emerin_regulatory  MASH1_promoter-coactivator  CAMK2-delta-MASH1_promoter-coactivator  Emerin_1  UTM-SGCE-DAG1-CAV1-NOS3  Sarcoglycan-sarcospan_SG-SPN  Rb-tal-1-E2A-Lmo2-Ldb1  TRP1-G_alpha-11-IP3R3-CAV1_signaling  ZO1-cadherin-VEGFR2  E-box_sequence-binding  Multisubunit_ACTR_coactivator  ILK-PARVB-ARHGEF6  NOS3-CAV1-NOSTRIN  NRP1-VEGFD  CAV1-VDAC1-ESR1  CFTR-NHERF-betaAR_signaling  ITGA9-ITGB1-FIGF  Class_C_Vps  ITGAV-ITGB3-PPAP2b  BKCA-beta2AR-AKAP79_signaling  PAR-6-PAR-3-VE-cadherin  AXIN-MEKK4-CCD1  PAR-3-VE-cadherin-beta-catenin  Chromatin_remodeling  ITGA4-ITGB1-JAM2  FOXO3-PCAF  OGT-TRAK1-TRAK2  NRP2-VEGFD  PCAF  CRLR-RAMP3  PARVB-ARHGEF6  p300/CBP-PCAF-MyoD  BKCA-beta2AR  HTR1A-EDG1  PAR-6-VE-cadherin  EDG1-HTR1D  eNOS-CAV1  CRLR-RAMP2  RNA_polymerase_II  ADRB2_homodimer  Caveolin-1_homodimer |
| m241 | PA700-20S-PA28  26S_proteasome  PA28-20S_proteasome  PA28gamma-20S_proteasome  20S_proteasome  Conserved_oligomeric_Golgi  COG1-COG8-COG2-COG3-COG4_subcomplex  COG1-COG8-COG5-COG6-COG7_subcomplex  Tetrameric_COG_subcomplex  COG1-COG8_subcomplex  COG |
| m27 | Nop56p-associated_pre-rRNA  PA700-20S-PA28  PA700  55S_ribosome  DNA_synthesome_core  CCT_micro-complex  LSm1-7  LSm2-8  PCNA-DNA_polymerase_delta  GINS  EIF3_core  28S_ribosomal_subunit  eIF3  EIF3  DNA_synthesome |
| m280 | Nop56p-associated_pre-rRNA  Ribosome  60S_ribosomal_subunit  TRBP_containing |
| m316 | GALNS-lysosomal_hydrolase_1.27_MDa |
| m340 | 55S_ribosome  28S_ribosomal_subunit  18S_U11/U12_snRNP  12S_U11_snRNP |
| m379 | SMCC  PC2  MED18-MED20-MED29_mediator_subcomplex  Mediator |
| m39 | 39S_ribosomal_subunit  28S_ribosomal_subunit  55S_ribosome  p27-cyclinE-Cdk2_-_Ubiquitin_E3_ligase  Ubiquitin_E3_ligase  CDH1-CKS1B  Oligosaccharyltransferase |
| m46 | CEN  CENP-A_NAC-CAD  BRCA1-BARD1-BACH1-DNA_damage_II  DNMT3B  BRCA1-BARD1-BACH1-DNA_damage_I  CENP-A_nucleosome_associated  Ubiquitin_E3_ligase  BARD1-BRCA1-CSTF  MCM2-MCM4-MCM6-MCM7  BRCA1_C  NDC80_kinetochore  BRCA1_A  GINS  P53-BARD1-Ku70  BRCA1-BARD1-UbcH5c  MCM4-MCM6-MCM7  BARD1-BRCA1-CSTF64  BRCA1_B  BRCA1-BARD1-BRCA2-DNA_damage_III  MCM2-MCM6-MCM7  BRCA1-BARD1-UbcH7c  BRCA1-BARD1-POLR2A  HUIC  MCM |
| m61 | Tacc1-chTOG-AuroraA |
| m62 | CEN  CDC5L  Emerin_24  RC_during_G2/M-phase_of_cell_cycle  Kaiso-NCOR  CTCF-nucleophosmin-PARP-HIS-KPNA-LMNA-TOP  Toposome  Condensin_I-PARP-1-XRCC1  MCM  Cell_cycle_kinase_CDC2  13S_condensin  MCM2-MCM4-MCM6-MCM7  RalBP1-CCNB1-AP2A-NUMB-EPN1  CDC2-PCNA-CCNB1-GADD45A  CDC2-PCNA-CCNB1-GADD45B  CDC2-PCNA-CCNB1-GADD45G  RalBP1-CDC2-CCNB1  MCM4-MCM6-MCM7  CDC2-CCNB1-CCNF  CDC2-CCNB1-PTCH1  CCNB1-CDC2  Condensin_I |
| m63 | LRP-1-Alpha-2-M-annexin_VI |
| m72 | TNF-alpha/NF-kappa_B_signaling_5  Cell_cycle_kinase_CDC2  MAD1L1-MAD2L1  MAD2-CDC20  CCNB2-CDC2  Mitotic_checkpoint |
| m82 | PA700-20S-PA28  26S_proteasome  PA28-20S_proteasome  PA28gamma-20S_proteasome  20S_proteasome  DNA-PK-Ku-eIF2-NF90-NF45 |
