# Supplementary material for: Cancer-Risk Module Identification and Module-Based Disease Risk Evaluation: A Case Study on Lung Cancer
Source: PLoS One. 2014 Mar 18;9(3):e92395. doi: 10.1371/journal.pone.0092395 (PMC3958511; doi:10.1371/journal.pone.0092395)
Supplement: Table S3 — The cancer-risk modules in the other four cancers. (DOC) [file pone.0092395.s003.doc]

Table S3 The cancer-risk modules in the other four cancers

| Profiles | Modules | Size | Genes |
| --- | --- | --- | --- |
| GSE14520  (Liver Cancer) | m116 | 4 | H3F3A*,H3F3AP4*,H3F3AP6,LOC730740*, |
|  | m126 | 3 | MTR*,ARID4B*,TSNAX*, |
|  | m136 | 3 | CTNNB1*,BOLA2*,BOLA2B*, |
|  | m146 | 3 | ETFDH*,BCKDHB*,PINK1*, |
|  | m154 | 3 | HTATSF1*,PSMD10*,VBP1*, |
|  | m163 | 3 | PSMD14*,PSMD1*,PSMD2*, |
|  | m176 | 3 | LOC728160*,UGT2B15*,UGT2B17*, |
|  | m19 | 17 | COPS5*,MRPL13*,EIF3E*,ZNF706*,AZIN1*,ATP6V1C1*,  POLR2K*,TMEM70*,ARMC1*,ENY2*,PEX2*,RPL30*,RPL7P52*,  TCEB1*,EIF3H*,EMC2*,KIAA0196*, |
|  | m28 | 11 | HNRNPR*,TOPBP1*,ATF2*,CD2AP*,PCNA*,RCN2*,SP3*,  TOP2B*,USP1*,DEK*,ANP32E*, |
|  | m31 | 10 | CHST4*,C1QTNF1*,DCN*,DPT*,PAMR1*,GGT5*,MFAP4*,  OLFML3*,C7*,SRPX*, |
|  | m52 | 7 | NME1*,NME2*,LRRC59*,NME1-NME2*,TKT*,TXNRD1*,PIR*, |
|  | m84 | 4 | CLIC1*,MAPRE1*,LAMC1*,CHMP3*, |
|  | m87 | 4 | SMNDC1*,SMC3*,BUB3*,VPS26A*, |
|  | m99 | 4 | ADSL*,ATIC*,NHP2L1*,EIF3D*, |
| GSE15781  (Colon Cancer) | m108 | 8 | B3GALT6*,COMT,GPS2,POLR2G,SMYD2,MRPL17*,  DCUN1D5*,PDCD5*, |
|  | m120 | 7 | CLUAP1*,GRB10*,DEXI,DDX19A*,PJA1*,TAF6,MSANTD4, |
|  | m123 | 7 | CD3EAP,STRAP,ARMC10P1,NAA38,TTC27,NARS2,ARMC10*, |
|  | m146 | 6 | GTF2F2,ICT1,MRPS23*,ZNF239,DTD1,TGS1*, |
|  | m156 | 6 | PTRH1,ADSL,TIMM9*,APEX1,POLR2F,LYRM4*, |
|  | m166 | 5 | AARS*,MTHFD1L*,MARS,ABCC1*,THUMPD2*, |
|  | m178 | 5 | PAIP1*,TRIM37,ZFR,IL33,MRPL33, |
|  | m195 | 5 | CCDC59,LOC440106,PHKA1,C2ORF49*,C12ORF29, |
|  | m214 | 4 | CACYBP,PFDN4*,OGFOD1*,TMEM126B, |
|  | m227 | 4 | HTRA2,RABL5*,SRPK2*,POMGNT2, |
|  | m255 | 4 | KIF3A,SENP6,ZNHIT6*,SPIRE1, |
|  | m290 | 4 | ABCB6*,CBFB,TAF1B,SART3, |
|  | m291 | 4 | GLA,YEATS2*,MAP2K1,ANP32E*, |
|  | m294 | 4 | DARS*,CIZ1*,ZDHHC4*,IMMP2L, |
|  | m322 | 3 | PLA2G16*,TBCB,WWOX, |
|  | m323 | 3 | NT5DC2,TTYH3*,GSTO1*, |
|  | m348 | 3 | FDXR,TRIAP1*,AEN*, |
|  | m356 | 3 | C12ORF45,DDX47*,LYAR*, |
|  | m365 | 3 | SLCO4A1,POLB*,IFITM1*, |
|  | m397 | 3 | SAE1,CTPS1*,SKA2, |
|  | m398 | 3 | INSL5*,GCG,PYY*, |
|  | m399 | 3 | SLC29A1,HSF1,EIF2AK3*, |
|  | m407 | 3 | OSTCP1,OSTC,RCN1*, |
|  | m438 | 3 | ABCA8,STMN2*,ADAMDEC1*, |
|  | m78 | 10 | SUGT1,EXOSC8,PHGDH,GTF3A*,NUP43,PGM2*,GPALPP1,  DDX27,NAA16,RNASEH2B, |
|  | m93 | 9 | ADAMTS19,OR10H2,PSAT1,IGF2,FAM89A*,C11ORF43,  SCD,MMP27,TYRO3, |
|  | m95 | 9 | CHGA*,CRYBA2*,PYY2*,PCSK1N*,NEUROD1,FEV*,SST,  TTR,MS4A8, |
| GSE20437  (Breast Cancer) | m160 | 3 | MID2,KAT7,RARS*, |
|  | m27 | 15 | TRIB1,EGR1,TIPARP,H3F3B,JUN,MCL1,ATF3,NR4A2*,  SNRK,RPGR,BTG2*,AHNAK,IER3,VAPA,IER2*, |
|  | m4 | 69 | RCAN2,CRISP3,ST3GAL6,TACC2,CFTR,STMN2,AHNAK2,  ADCY8,CKB,CKMT1B,CKMT2,CYP3A7,CYP3A5,AKR1C1,  AKR1C2,DSG1,SLC26A2,EXTL1,F5,BICD2*,QPCT,CNPPD1,  GOT1,KRT6C,GRB14,GRIA2,HSD11B2,HYAL1,IL13RA2,AQP5,  INHBA,KRT6A,KRT6B,KRT16,LGALS7,MAL,MGAT3,  CEACAM6,NEUROD1,ATP1B3,PAEP,SERPINB2,PCP4,  PDHA1,PLCB4,CKMT1A,PTGFR,S100A2,S100P,PINK1,  LGALS7B,SLC9A2,SLC14A1,TG,TSPAN8,CRISP2,WNT11,  CA6,GNPTAB,HOPX,DNAH17,RUNX3,SCEL,CLDN10,SYNGR3,  NR1D1,RAPGEF2,CDA,KIAA0319, |
| GSE26126  (Prostate Cancer) | m26 | 14 | UBE2E3*,DFFB*,SMG5*,UBE2S*,SH3KBP1*,APBB2*,  ZFYVE1*,PMS2*,PREP*,HSPA13*,UGDH*,RBM4B*,  PDIA4*,MTL5*, |
|  | m27 | 12 | USP54*,DMBT1*,GPLD1*,KLK3*,ZBTB7B*,CDH26*,  SLC4A1*,THOC6*,BPIFB2*,RGS9*,MPZL1*,KIAA0355*, |
|  | m51 | 4 | CYYR1*,CBLN4*,FGF10*,MAGI2*, |
|  | m60 | 3 | SPATA12*,PNPLA2*,BBOX1*, |
|  | m61 | 3 | KCNE3*,CORIN*,CYP27A1*, |
|  | m66 | 3 | ITGA5*,WT1-AS*,WT1*, |
|  | m73 | 3 | SLC39A1*,ELOVL6*,E2F8*, |
